# Downregulation of miR-223 promotes HMGB2 expression and induces oxidative stress to activate JNK and promote autophagy in an *in vitro* model of acute lung injury

**DOI:** 10.1186/s12950-021-00295-3

**Published:** 2021-11-03

**Authors:** Hao-Yu Tan, Bei Qing, Xian-Mei Luo, Heng-Xing Liang

**Affiliations:** 1 Department of Cardio-vascular Surgery, the Second Xiangya Hospital of Central South University, No.139 Middle Renmin Road, Hunan Province 410011 Changsha, People’s Republic of China; 2 Department of Thoracic Surgery, the Second Xiangya Hospital of Central South University, No.139 Middle Renmin Road, Hunan Province 410011 Changsha, People’s Republic of China

**Keywords:** Autophagy, HMGB2, MiR-223, Oxidative stress, JNK signalling

## Abstract

**Background:**

Excessive autophagic activity in alveolar epithelial cells is one of the main causes of acute lung injury (ALI), but the underlying molecular mechanism has not been fully elucidated. Previous studies have shown that microRNAs (miRs) are involved in regulating autophagy in several diseases. This study aimed to determine the role of miR-223 in excessive autophagic activity in alveolar epithelial cells and the underlying mechanism to identify a novel therapeutic targets for the development of new drugs to treat acute respiratory distress syndrome (ARDS).

**Methods:**

A549 cells were treated with lipopolysaccharide (LPS) to establish an ALI *in vitro* model. The expression of miR-223 and its role of miR-223 in regulating oxidative stress and autophagy in the LPS-treated A549 cells, were examined using RT-PCR, flow cytometry and ELISA. A luciferase reporter assay was performed to verify the interaction between miR-223 and the high-mobility group box 2 (HMGB2) protein.

**Results:**

The results showed that the LPS treatment downregulated miR-223 expression in alveolar epithelial cells. We further proved that miR-223 directly targeted the 3-untranslated region of the HMGB2 gene and the downregulation of miR-223 increased HMGB2 protein level, which activated the JNK signalling pathway and thus induced oxidative stress and autophagy in LPS-treated alveolar epithelial cells. Knockdown of HMGB2 protein deactivated the JNK signalling pathway and inhibited autophagy and oxidative stress in alveolar epithelial cells.

**Conclusions:**

The results of this study suggest that miR-223 regulates oxidative stress and autophagy in alveolar epithelial cells by targeting HMGB2 via the JNK signalling pathway.

**Supplementary Information:**

The online version contains supplementary material available at 10.1186/s12950-021-00295-3.

## Background

Acute respiratory distress syndrome (ARDS) is manifested in acute lung injury (ALI) [1]. The average mortality rate of ARDS can be as high as approximately 30 % [1, 2]. There are no effective therapeutic drugs in clinical practice for ARDS, and supportive treatments remain to be the principal treatment options [1]. ALI is caused by various factors, such as trauma and infection [1]. Past studies have shown that autophagy was an induced reaction to the injury [3, 4]. Excessive induction of autophagy in alveolar epithelial cells can increase the secretion of inflammatory factor secretion and cell death, which resulting in aggravation of ARDS [3, 5]. Thus, understanding the autophagic regulation mechanism during ALI is crucial.

The high-mobility group box (HMGB) protein family is related to the development of inflammatory diseases, including myocardial ischaemia and ARDS [6–8]. According to a previous study, the HMGB2 level in serum was proportional to myocardial infarction severity [6]. HMGB2 enhanced reactive oxygen species (ROS) production and led to abnormal cell apoptosis, inflammation, and autophagic activity in cardiac cells [6]. A recently published study revealed that HMGB2 expression was abnormally increased in the plasma and lung epithelial lining fluid of ALI patients [9]. These results may indicate that HMGB2 potentially plays a role in regulating ALI, but further studies are needed to confirm this hypothesis.

MiRNAs (miRs) are the small single noncoding RNA molecules involved in regulating many diseases and post-transcriptionally mediating specific targeted mRNAs’ expression [3, 10, 11]. It has been reported that miR-455 interacts with the long noncoding RNA GAS5 and regulates M1 macrophage polarizationpromote in childhood pneumonia [12]. Another study documented that the low miR-223 expression was associated with severe lung inflammation and excessive autophagic activity in alveolar epithelial cells [13, 14]. However, the functional and regulatory roles of miR-223 in ALI and the downstream targets of miR-223 in ALI still need to be elucidated to understand its underlying mechanism.

Signalling pathways, including the nuclear factor-κB (NF-κB) and phosphatidylinositol 3-kinase (PI3K), and mitogen-activated protein kinase (MAPK) pathways, were upregulated in animal models of ALI [15–17]. C-Jun NH2-terminal kinase (JNK), a member of the MAPK family, was identified to play an important role in regulating ALI [7, 18, 19]. Furthermore, the JNK inhibitor, SP600125, has a protective effect on ALI *in vivo* and *in vitro* [7, 18].

Based on the above evidence, we hypothesized that miR-223 mediated lung injury by regulating HMGB2 via the JNK signaling pathway. The increased HMGB2 in ALI patients induced ROS production, thus activated the JNK signaling pathway and aggravated cellular autophagic activity.

## Materials and methods

### Cell culture and induction of cell injury using LPS

Human alveolar epithelial cells (A549, American Type Culture Collection (ATCC)) were cultured and maintained according to the manufacturer’s instructions and in the appropriate media. Under control conditions, A549 cells were treated with LPS (5, 10 and 20 µg/mL) for 6, 12, 24, 48 and 72 h, respectively. Then, A549 cells treated with different LPS concentrations for different times points were harvested for the MTT assay. Only A549 cells treated with LPS for 72 h were analysed with quantitative polymerase chain reaction (qPCR), western blot, and enzyme-linked immunosorbent assay (ELISA).

### MTT assay

The viabilities of A549 cells treated with different LPS concentrations of LPS for different times and untreated A549 cells were evaluated by MTT assay according to the manufacturer’s instructions. Briefly, approximately 1 × 10^5^ mL^−1^ LPS-treated A549 cells or untreated A549 cells were seeded in flat-bottomed 96-well polystyrene coated plates. After 24 h of incubation, 10 µL of MTT reagent was added to each well, followed by incubation for another 4 h. The plates were then read immediately in a microplate reader (BIO-RAD microplate reader-550) at 570 nm.

### ELISA

ELISA kits for 8-hydroxy-2’-deoxyguanosine (8-OHdG), tumour necrosis factor α (TNFα), interleukin-1β (IL-1β) and interleukin 6 (IL-6) were utilized according to the manufacturer’s instructions. 100 µL of the 8-alveolar epithelial cells medium were tested per well. Values were assayed in triplicate and calibrated against an 8-OHdG standard.

### Western blot

Proteins were isolated with 12 % SDS–PAGE and then transferred onto homopolymers and copolymer membranes (Schleicher & Schuell, Germany). The membranes were blocked in phosphate-buffered saline (PBS) that containing 10 % nonfat dry milk and 0.5 % Tween-20 overnight. Subsequently, the membranes were incubated with primary antibodies for 2 h. The following antibodies were used: anti- LC3B (Cell Signaling Technology, Danvers, MA, USA; #2775; 1:1000), anti-p62 (Cell Signaling Technology; #5114; 1:1000), anti-HMGB2(Abcam, Cambridge Science Park, UK; ab67282; 1:1000), p-JNK (Abcam, Cambridge Science Park, UK; ab47337; 1:1000), JNK (Abcam, Cambridge Science Park, UK; ab213521; 1:1000), and anti-β-actin (Abcam, Cambridge Science Park, UK; ab8226; 1:1000). The bands were visualized using a chemiluminescence detection system (CWBIO; Beijing, China) and were normalized to that of β-actin.

### ROS assay

CellROX™ Deep Red (Invitrogen, USA) was used to measure intracellular ROS production. CellROX® Deep Red reagent was added at a final concentration of 5 µM to human alveolar epithelial cells, followed by incubation 37 °C for 30 min. The medium was removed, and the cells were washed three times with PBS. The fluorescence was measured at 644 nm excitation and 665 nm wavelengths utilizing a Bio-Tek Synergy HT-I plate reader (Bio-Tek Instruments, USA).

### RT-qPCR

Total RNA was isolated from A549 cells using TRIzol (Invitrogen). Then RNA was reverse transcribed using a PrimeScript™ RT reagent kit (cat. No. RR037A; Takara Biotechnology Co. Ltd.). RT-qPCR was performed using RT-qPCR UltraMix(SYBR Green) (LMAI Bio) according to the manufacturer’s instructions. Each reaction was performed in triplicate. Relative expression levels were calculated by the 2^−∆∆Ct^ method and normalized to those of the GAPDH gene and U6. The primers were as follows: HMGB2: Forward, 5’-GTGGCCTAGCTCGTCAAGTT-3’; Reverse, 5’-GCGTACGAGGACATTTTGCC-3’; miR-223: Forward, 5’-GGCGCTTGTCAGTTTGTCAAAT-3’; Reverse, 5’-GTCGTATCCAGTGCAGGGTCCG-3’; GAPDH: Forward, 5’-CCAGGTGGTCTCCTCTGA-3’; Reverse, 5’-CCGTGTTCCTACCCCCAATG-3’; U6: Forward, 5’-GCTTCGGCAGCACATATACTAAAAT-3’; Reverse, 5’-CGCTTCACGAATTTGCGTGTCAT-3’.

### Flow cytometry

A549 cells were trypsinized and washed twice with PBS. The cells were stained with Annexin V and propidium iodide (PI) using an FITC Annexin V/PI Apoptosis Detection Kit (BD Biosciences, San Jose, CA, USA) based on the manufacturer’s instructions. Briefly, cells were seeded in 24-well plates for approximately 24 h and then stained with the Annexin V-FITC Annexin V and PI solution for 15 min. Then, the cells were analysed using a FACS cytometer (BD Biosciences).

### Construction of the plasmid vector and cell transfection

The HMGB2 gene and pcDNA3.1 vector were used to construct an RNA knockout vector. MiR-223 mimic and inhibitor and their negative control oligonucleotides were obtained from GenePharma (Shanghai, China). The JNK inhibitor, SP600125, was purchased from Calbiochem (San Diego, CA). According to the manufacturer’s instruction, cells were transfected using LipofectamineTM 2000 (Invitrogen, Carlsbad, USA). Briefly, 10 µg/mL alveolar epithelial cells were seeded to flasks until they were 70 % - 80 % confluent. Then, cells were transfected with 20 µL Lipofectamine followed by rinsing with the serum-free, antibiotic-free medium. After 6 h of incubation, the transfected cells were resuspended and cultured in the regular cell culture medium for 72 h before analyses. Then the cells were treated with SP600125 (20 µg/mL) or LPS (10 µg/mL) to induce injury.

### Luciferase assay

Site-directed mutagenesis of the miR-223 target site at the 3’-UTR of HMGB2 was performed utilizing a QuickChange mutagenesis kit (Stratagene, Heidelberg, Germany). HMGB2 wild-type (wt-HMGB2) or mutant (mut-HMGB2) 3’-UTR nucleotide sequences were inserted into the pLuc luciferase vector (Ambion, Austen, Texas, USA). Alveolar epithelial cells, which were cultured in 24-well plates, were transfected with 100 ng of wt-HMGB2 or mut-HMGB2 and 50 nM of miR-223 mimic or miR-223 inhibitor utilizing Lipofectamine™ 2000 (Invitrogen). The cells were harvested following 48 h after the transfection. According to the manufacturer’s instructions, a Dual-Luciferase Reporter assay kit was used to measure luciferase activity (Promega, Madison, Wisconsin, USA). All transfections were performed in triplicates.

### Statistical analysis

All statistical analyses were performed using GraphPad Prism 5.0. Two-sample t-tests were used for comparisons between two groups. In cases of comparison among more than two groups, ANOVA was applied. Each experiment was performed at least three times independently with cells from three different passages. Data were presented as the mean ± standard deviation (SD). A value of P < 0.05 was considered statistically significant.

## Results

### LPS-induced oxidative stress and excessive autophagic activity in alveolar epithelial cells

**Fig. 1:**
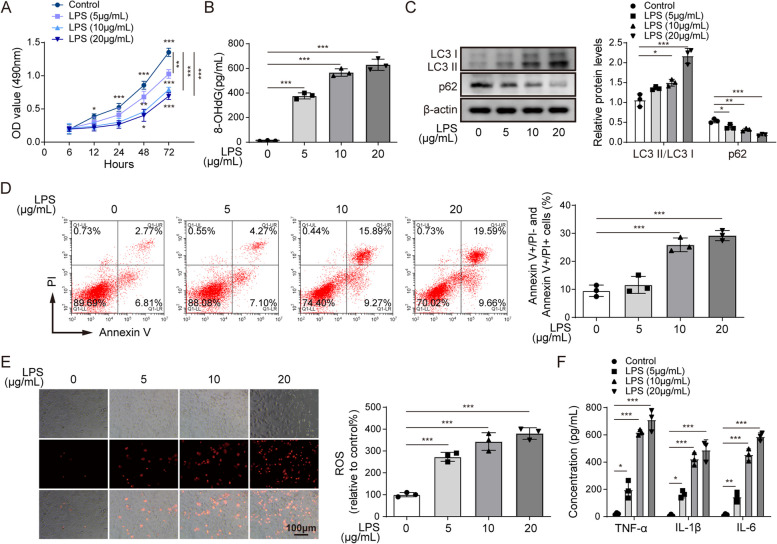
LPS induced oxidative stress and autophagy in alveolar epithelial cells. **A** MTT assay the effects of different concentrations of LPS on A549 cell viability between 6 and 72 h. **B** Changes in 8-OHdG expression induced by different concentrations of LPS in A549 cells were detected by ELISA. **C** Changes in autophagy-related proteins induced by LPS at different concentrations in A549 cells were detected by western blot. **D** Changes in the apoptotic rate of A549 cells treated with LPS at different concentrations were assessed via flow cytometry. **E** The fluorescent probe detected the changes in the ROS level in A549 cells induced by different concentrations of LPS. **F** The concentrations of TNF-α, IL-1β and IL-6 in A549 cells treated with different concentrations were assessed via ELISA. *n *=3. *P < 0.05, ** *P* < 0.01, *** *P* < 0.001

A549 cells were treated with 5, 10 and 20 µg/mL of LPS for 6, 12, 24, 48 and 72 h, and then oxidative stress and autophagic activity in the cells were analysed. Figure 1A indicated that the cell viability was decreased after LPS treatment and that the impact of LPS on the A549 cell vitality was time-dependent and concentration-dependent. 72 h of treatment with 10 µg/mL LPS induced the most significant decrease in cell viability (two-way analysis of variance). When treated with 20 µg/mL LPS, A549 cells showed the lowest viability rate (two-way analysis of variance). To further understand the impact of LPS with different concentrations on oxidative stress and excessive autophagic activity in alveolar epithelial cells, we treated A549 cells with 5, 10 and 20 µg/mL of LPS for 72 h. The concentration of 8-OHdG, a critical biomarker of oxidative stress, was significantly increased upon LPS treatment in a concentration-dependent manner (Fig. 1B, one-way ANOVA). Similarly, LPS significantly decreased p62 expression but increased the conversion of LC3I to LC3II in a concentration-dependent manner (Fig. 1C, one-way ANOVA). Apoptotic cells were also increased after LPS treatment. Compared with control treatment and 5 µg/mL LPS treatment, 10 and 20 µg/mL LPS treatment induced the highest percentages of apoptotic cells, and no significant difference was found between the 10 and 20 µg/mL LPS treatment groups (Fig. 1D, one-way ANOVA). The ROS levels in the LPS-treated cells were detected by a fluorescent probe (Fig. 1E). The cells treated with 10 and 20 µg/mL LPS showed a significantly increased ROS levels compared to the control and 5 µg/mL LPS-treated cells (one-way ANOVA). Furthermore, the LPS treatment significantly induced the expressions of inflammatory-related factors, including TNF-α, IL-1β and IL-6 expressions, in a concentration-dependent manner (Fig. 1F, one-way ANOVA).

Overall, we found that compared with the control and 5 µg/mL LPS treatment groups, the 10 µg/mL LPS treatment group showed the most significantly altered ratio, with no significant difference compared to the 20 µg/mL LPS treatment group. These findings indicate that 10 µg/mL LPS treatment can significantly induce oxidative stress and autophagy in alveolar epithelial cells.

### The miR-223 suppresses oxidative stress and autophagy in LPS-treated alveolar epithelial cells

**Fig. 2:**
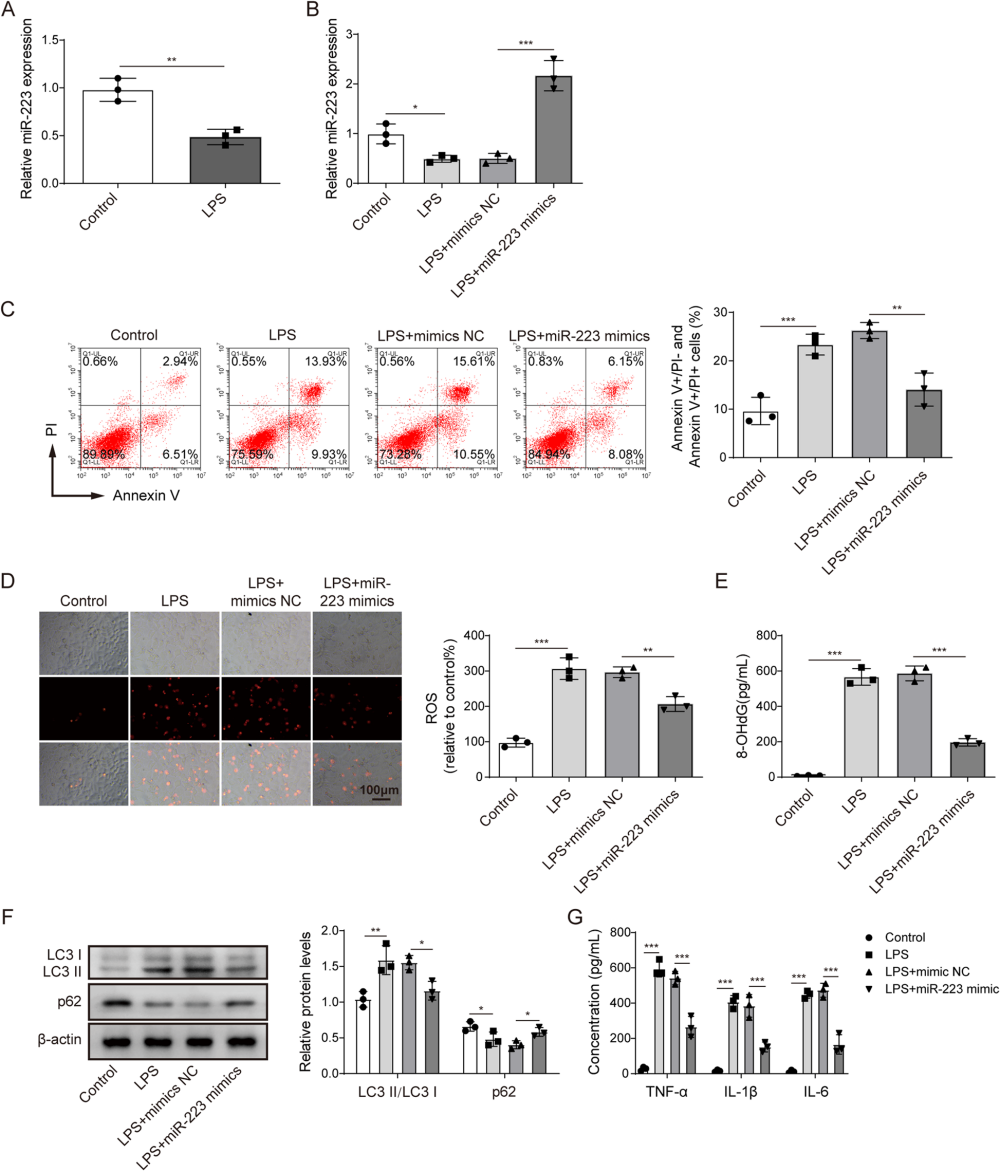
The miR-223 suppresses oxidative stress and autophagy in LPS-treated alveolar epithelial cells.** A** RT-qPCR detection of the miR-223 expression in LPS-treated A549 cells. **B** After A549 cells were transfected with the miR-223 mimic, the cells were further induced by LPS. The miR-223 expression was detected by RT-qPCR. **C** The effect of miR-223 overexpression plus LPS treatment or LPC treatment on A549 cell apoptosis was assessed by flow cytometry. **D** The fluorescent probe was used to detect ROS level in A549 cells following treatment with LPS in the presence or absence of miR-223 overexpression. **E** The 8-OHdG concentration in A549 cells following treatment of LPS in the presence or absence of miR-223 overexpression was assessed by ELISA. **F** Western blot analyses of the autophagy-related proteins in A549 cells following treatment with LPS in the presence or absence of miR-223 overexpression. **G** The concentrations of TNF-α, IL-1β and IL-6 in A549 cells following LPS treatment in the presence or absence of miR-223 overexpression were assessed by ELISA. *n*=3. *P < 0.05, ** *P* < 0.01, *** *P* < 0.001

Next, we investigated whether miR-223 was associated with the excessive autophagy in alveolar epithelial cells. The gene expression of miR-223 was significantly decreased in the LPS-treated A549 cells compared to the control cells (Fig. 2A, Student t-test). However, the expression of miR-223 was restored in LPS-treated A549 cells after transfection with miR-223 mimics (Fig. 2B, one-way ANOVA). Overexpression of miR-223 reversed LPS-treated A549 cell apoptosis and ROS level in A549 cells (Fig. 2C and D, one-way ANOVA). ELISA results showed that miR-223 overexpression reversed the LPS-treated increase in the 8-OHdG concentration in A549 cells (Fig. 2E, one-way ANOVA). Furthermore, the ratio of LC3II to LC3I in LPS-treated A549 cells was downregulated following miR-223 mimic transfection. In contrast, the level of p62 protein in LPS-treated A549 cells was upregulated following miR-223 mimic transfection (Fig. 2F, one-way ANOVA). miR-223 overexpression suppressed the LPS-induced increase in inflammatory-related factors, including TNF-α, IL-1β and IL-6 (Fig. 2G, one-way ANOVA). In summary, the results showed that overexpression of miR-223 attenuates LPS-induced oxidative stress and autophagic activity in A549 cells.

### MiR-223 directly targets HMGB2 to regulate its expression

**Fig. 3:**
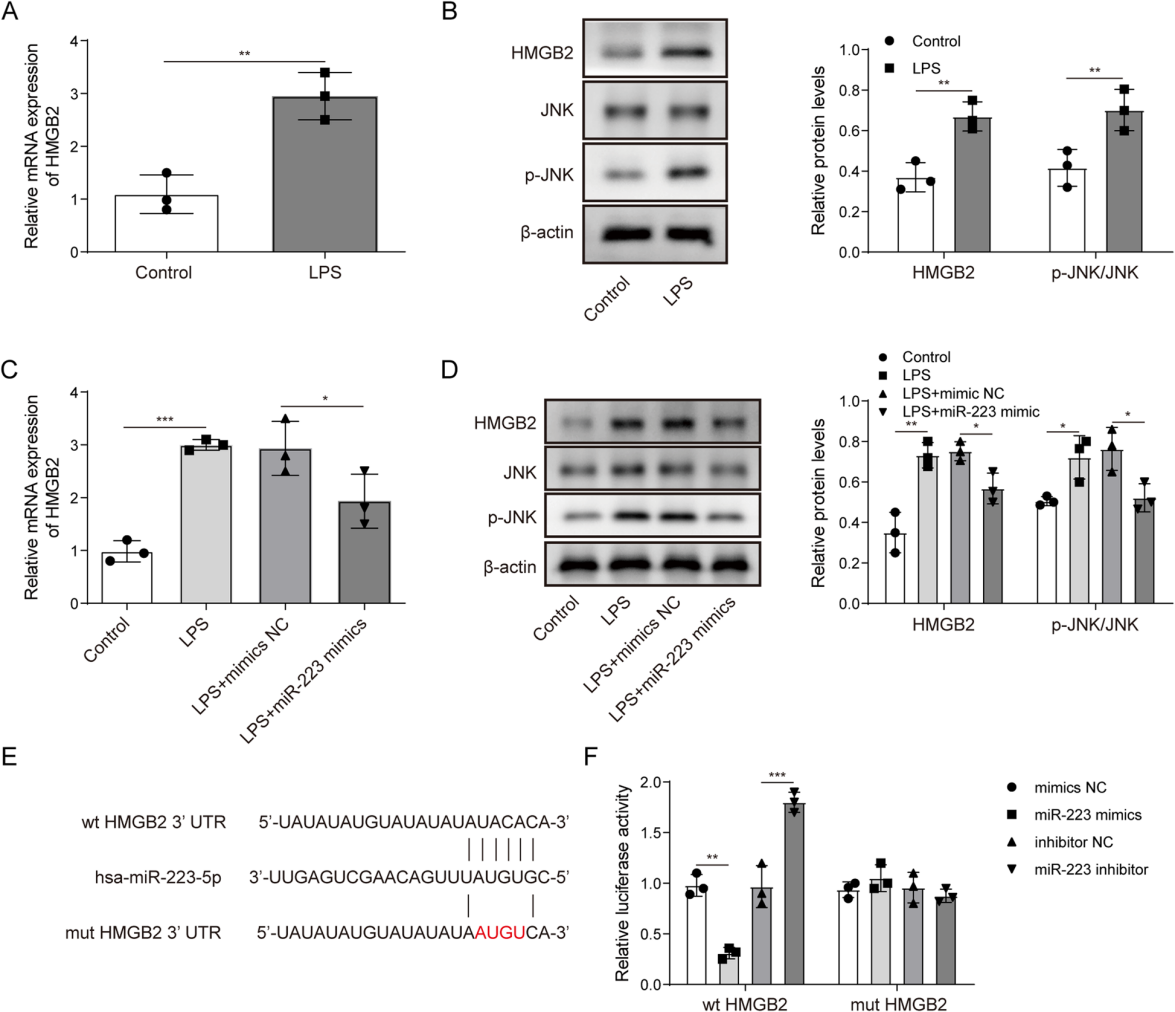
MiR-223 directly binds HMGB2 and regulates its expression. **A** RT-qPCR detected the gene expression of HMGB2 in the LPS-treated A549 cells and the untreated control cells. **B** Western blot analysis of HMGB2, JNK, p-JNK and β-actin in the LPS-treated A549 cells. **C** The effect of miR-223 overexpression plus LPS treatment or only LPS treatment on HMGB2 mRNA expression. **D** The expression of HMGB2, JNK, p-JNK and β-actin proteins in A549 cells following treatment with LPS in the presence or absence of miR-223 overexpression was detected by western blot. **E** Online prediction of miR-223 and HMGB2 binding sites. **F** The A549 cells were co-transfected with miR-223 mimics or a miR-223 inhibitor. Then, luciferase activity was detected. *n*=3. *P < 0.05, ** *P* < 0.01, *** *P* < 0.001

Using online prediction, we found one binding site of miR-223 in the 3’-UTR of HMGB2. Therefore, we speculated that miR-223 regulated autophagy potentially by targeting HMGB2. HMGB2 was significantly upregulated in the LPS-treated A549 cells compared to control cells (Fig. 3A, Student t-test). Western blot analysis revealed that the protein expression of HMGB2 and the phosphorylated JNK to JNK ratio were increased in the LPS-treated cells (Fig. 3B). However, miR-223 overexpression reversed the promotion effect of LPS on HMGB2 and the phosphorylated JNK to JNK ratio in A549 cells (Fig. 3C and D, one-way ANOVA). TargetScan7.2 was used to predict the potential complementary sequence between miR-223 and HMGB2 (Fig. 3E). MiR-223 mimics significantly decreased the luciferase activity of A549 cells transfected with wt-HMGB2, but not cells transfected with mut-HMGB2 (Fig. 3F, one-way ANOVA). Consistently, the miR-223 inhibitor significantly increased the luciferase activity of A549 cells transfected with wt-HMGB2, but not cells transfected with mut-HMGB2. Taken together, the miR-223 is directly bound to a specific site on the 3’-UTR of HMGB2 to regulate its expression.

### HMGB2 mediates LPS-induced autophagy of alveolar epithelial cells by stimulating ROS to produce oxidative stress and activating JNK signalling

**Fig. 4:**
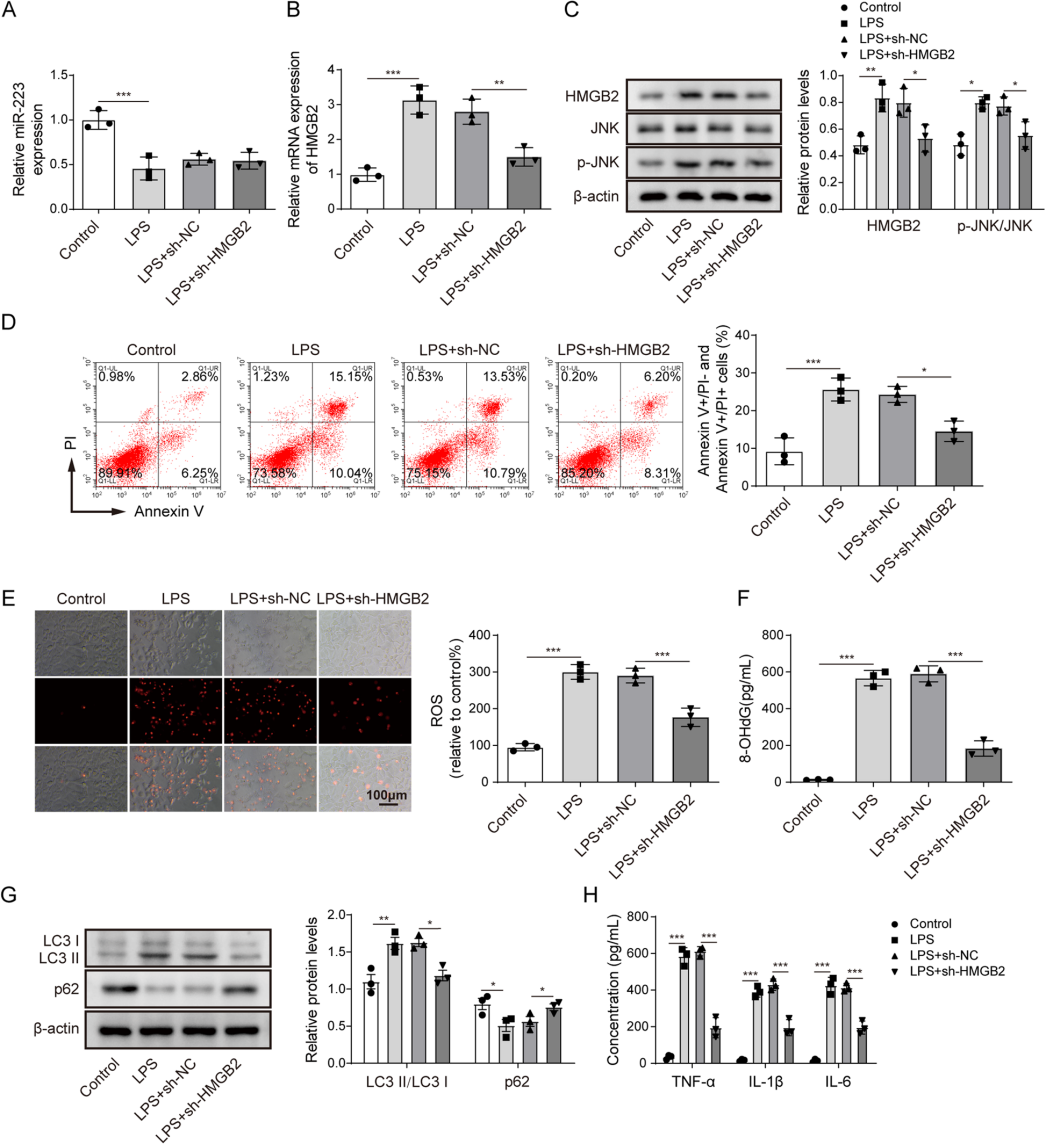

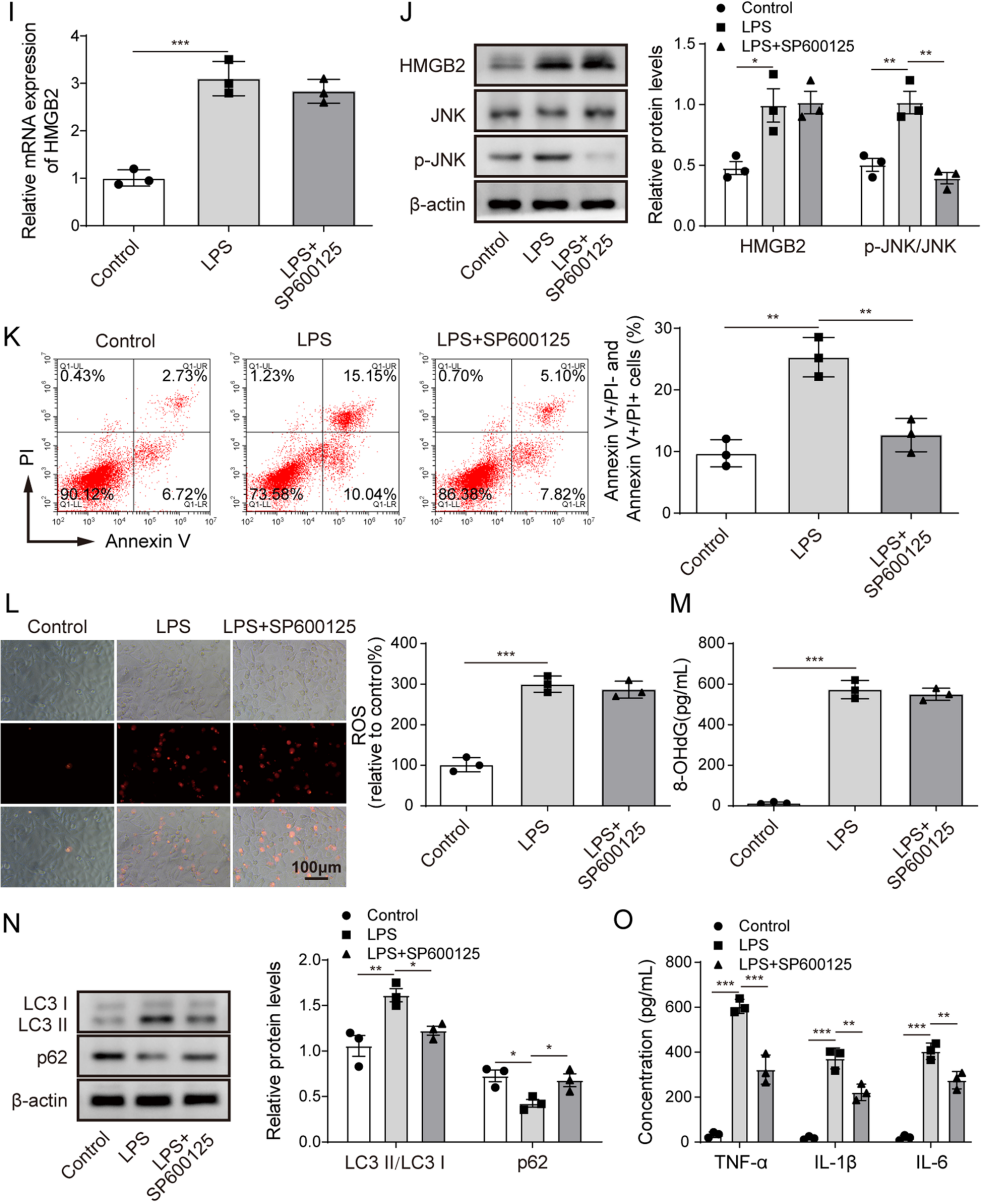
HMGB2 mediates LPS-induced autophagy of alveolar epithelial cells by stimulating ROS to produce oxidative stress and activating JNK signalling.** A** and **B** The expressions of miR-223 and HMGB2 in A549 cells transfected with sh-HMGB2 or sh-NC plus LPS were assessed by RT-qPCR. **C** Representative western blot results of HMGB2, JNK, p-JNK and β-actin expressions in A549 cells transfected with sh-HMGB2 or sh-NC plus LPS. **D** The effect of HMGB2 knockdown plus LPS treatment or LPS treatment on A549 cell apoptosis was assessed by flow cytometry. **E** The Fluorescent probe was used to detect the ROS level in A549 cells transfected with sh-HMGB2 or sh-NC plus LPS treatment. **F** The 8-OHdG concentrations in A549 cells transfected with sh-HMGB2 or sh-NC plus LPS treatment were assessed by ELISA. **G** Western blot analysis of the autophagy-related proteins in A549 cells transfected with sh-HMGB2 or sh-NC plus LPS treatment. **H** The concentrations of TNF-α, IL-1β and IL-6 in A549 cells transfected with sh-HMGB2 or sh-NC plus LPS treatment were assessed by ELISA. **I** The expression level of HMGB2 in A549 cells after LPS induction and the addition of PS600125 (20 μm/L) was detected by RT-qPCR. **J** HMGB2, JNK, p-JNK and β-actin protein levels in LPS induced A549 cells or LPS induced and PS600125 treated cells were assessed by western blot. **K** The effect of LPS treatment or LPS plus PS600125 treatments on A549 cell apoptosis was assessed by flow cytometry. **L** The Fluorescent probe was used to detect ROS level in LPS induced A549 cells or LPS-treated and PS600125 treated cells. **M** ELISA detected the concentrations of 8-OHdG in LPS induced A549 cells or LPS induced and PS600125 treated cells. **N** Western blot analysis of the autophagy-related proteins in LPS induced A549 cells or LPS induced and PS600125 treated cells. **O** The concentrations of TNF-α, IL-1β and IL-6 in LPS induced A549 cells or LPS induced and PS600125-treated cells were assessed by ELISA. *n*=3. *P < 0.05, ** *P* < 0.01, *** *P* < 0.001

To further confirm the role of HMGB2 in mediating oxidative stress and autophagy in LPS-treated alveolar epithelial cells, we treated the A549 cells with sh-HMGB2. Downregulation of HMGB2 had no significant impact on miR-223 expression in A549 cells (Fig. 4A, one-way ANOVA). However, knockdown of HMGB2 successfully reversed the LPS-treated increase in HMGB2 levels, as indicated in Fig. 4B and C (one-way ANOVA). Silencing of HMGB2 also reduced the LPS-induced the ratio of JNK phosphorylation to JNK in A549 cells, suggesting that HMGB2 knockdown inhibited the JNK signalling pathway. Knockdown of HMGB2 reversed the LPS-induced A549 cell apoptosis (Fig. 4D, one-way ANOVA). HMGB2 downregulation also attenuated LPS-induced increase in ROS levels and 8-OHdG concentrations in A549 cells (Fig. 4E F, one-way ANOVA). In addition, knockdown of HMGB2 suppressed the LC3II to LC3I ratio but promoted p62 expression in LPS-treated A549 cells (Fig. 4G, one-way ANOVA). The downregulation of HMGB2 decreased the promotion effect of LPS on inflammatory-related factors, including TNF-α, IL-1β and IL-6, in A549 cells (Fig. 4H, one-way ANOVA).

To further verify that HMGB2 regulated LPS-induced autophagy in alveolar epithelial cells via the JNK signalling pathway, we inhibited the JNK signalling pathway by adding SP600125, a JNK inhibitor [20]. Inhibition of the JNK signalling pathway did not induce any significant change in HMGB2 expression in A549 cells (Fig. 4I, one-way ANOVA). However, the SP600125 reduced the LPS-induced the ratio of phosphorylated JNK to JNK (Fig. 4J, one-way ANOVA). Treatment with SP600125 reversed LPS-induced A549 cell apoptosis (Fig. 4K, one-way ANOVA). The JNK inhibitor did not influence the ROS level or 8-OHdG concentration in A549 cells (Fig. 4L and M, one-way ANOVA). In contrast, inhibition of the JNK signalling pathway inhibited LPS-induced modification of the autophagy-related proteins and upregulation of the inflammatory-related proteins (Fig. 4N and O, one-way ANOVA). Taken together, HMGB2 mediates LPS-induced autophagy of alveolar epithelial cells by stimulating ROS to produce oxidative stress and activating JNK signalling.

### MiR-223 mediates oxidative stress and JNK signalling pathway activation by regulating HMGB2 expression, thereby affecting LPS-induced autophagy of alveolar epithelial cells

**Fig. 5:**
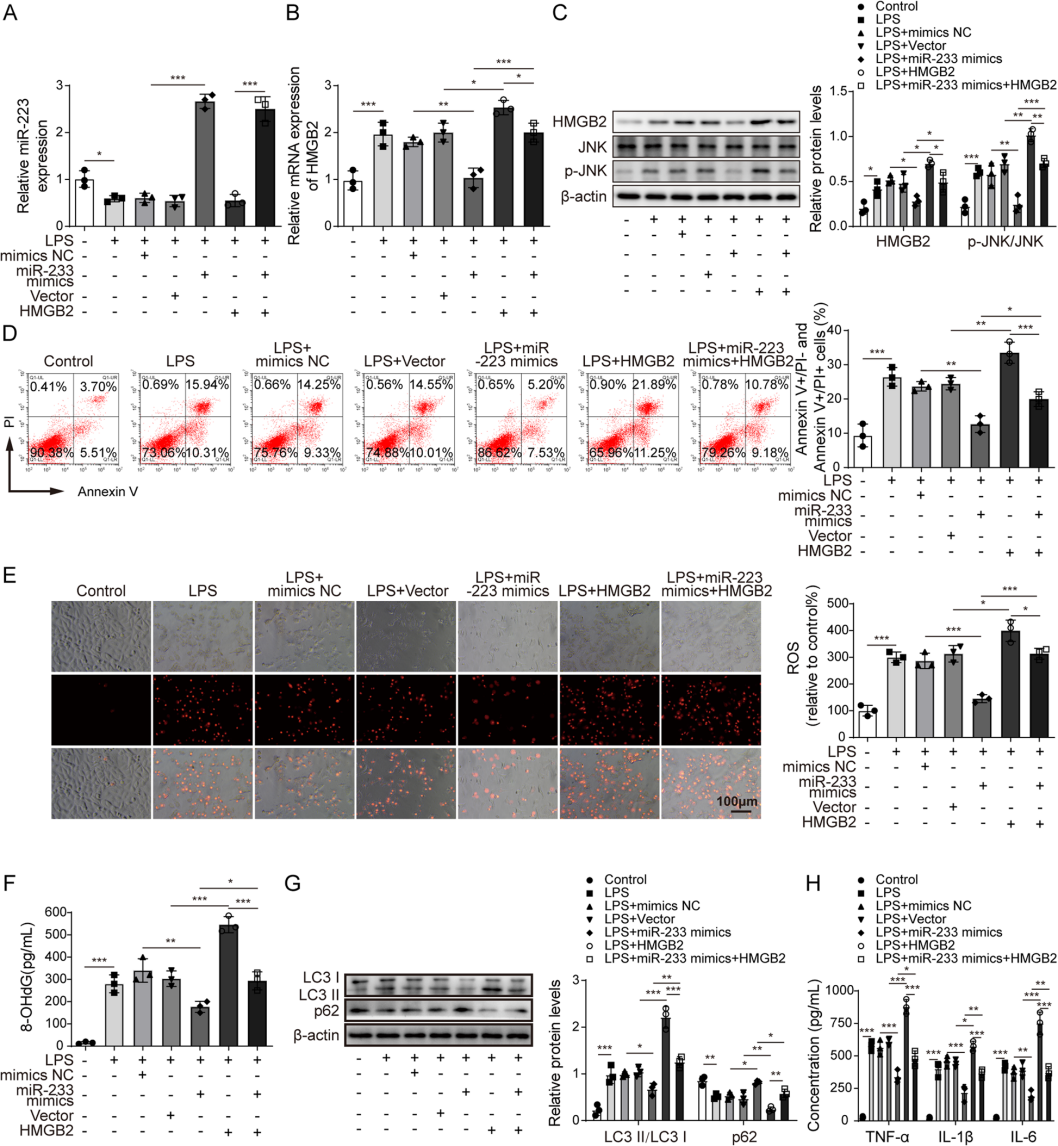
MiR-223 mediates oxidative stress and JNK signalling pathway activation by regulating HMGB2 expression, thereby affecting LPS-induced autophagy of alveolar epithelial cells. A549 cells were simultaneously transfected with miR-223 mimics and HMGB2 overexpression vectors and then incubated with LPS to induce injury. **A** The miR-223 expression in the cells was detected by RT-qPCR. **B** RT-qPCR was used to detect the mRNA expression of HMGB2. **C** Protein expression levels HMGB2, JNK, p-JNK, and β-actin in the cells were detected by western blot. **D** The apoptotic rate was assessed by flow cytometry and its quantification data. **E** The fluorescent probe detected ROS level in the cells. **F** ELISA was used to detect the concentration of 8-OHdG in cells. **G** Western blot analysis of the autophagy-related proteins in the cells. **H** The concentrations of TNF-α, IL-1β and IL-6 were assessed by ELISA. *n*=3. *P < 0.05, ** *P* < 0.01, *** *P* < 0.001

Finally, we examined the effect of simultaneous overexpression of miR-223 and HMGB2 on oxidative stress and autophagy in LPS-treated A549 cells. RT-qPCR results confirmed the overexpression of miR-223 in A549 cells, and overexpression of HMGB2 did not affect miR-223 expression (Fig. 5A). The gene and protein expression levels of HMGB2 were significantly reduced in the miR-223 overexpressed and LPS-treated cells. However, the expression of HMGB2 was restored by HMGB2 overexpression (Fig. 5B, one-way ANOVA). Overexpression of miR-223 significantly decreased the ratio of phosphorylated JNK to JNK in LPS-treated A549 cells, whereas overexpression of HMGB2 had the opposite effect (Fig. 5C, one-way ANOVA). HMGB2 overexpression restored the inhibitory effect of miR-223 overexpression on LPS-treated cell apoptosis (Fig. 5D, one-way ANOVA). Similarly, overexpression of HMGB2 reversed the inhibitory effect of miR-223 overexpression on LPS-treated increasing of ROS level and 8-OHdG concentration in A549 cells (Fig. 5E, and F, one-way ANOVA). Additionally, overexpression of HMGB2 attenuated miR-223 overexpression-induced downregulation of p62 and the conversion of LC3I to LC3II in LPS-treated A549 cells (Fig. 5G, one-way ANOVA). miR-223 overexpression reversed LPS-treated release of inflammatory factors, whereas the inhibitory effect of miR-223 overexpression was restored by the overexpression of HMGB2 (Fig. 5H, one-way ANOVA). In summary, miR-223 overexpression reduces HMGB2 expression and thus suppressed oxidative stress and excessive autophagy in LPS-treated alveolar epithelial cells. Nevertheless, the protective effect of miR-223 mimic was reversed by HMGB2 overexpression.

## Discussion

Previous studies have shown that in ALI, the autophagy in alveolar epithelial cells was excessively activated, which is one of the main causes of ALI [3, 21, 22]. Understanding the mechanism of alveolar epithelial cell autophagy can facilitate the discovery of new therapies for ALI treatment. This study revealed the critical role of miR-223 in regulating oxidative stress and autophagy in alveolar epithelial cells. We further demonstrated that miR-223 negatively regulates HMGB2, which mediates the JNK signalling pathway and ALI activation. To the best of our knowledge, this is the first time that the regulatory role of HMGB2 in ALI is reported.

Several studies have shown that miR-223 may act as an anti-inflammatory mRNA and is associated with lung inflammation and autophagy in lung cells [1, 23]. Yan et al. demonstrated that miR-223 was downregulated in the lipopolysaccharide-induced ALI model and the downregulated miR-223 promoted inflammation and the TLR4/NF-κB signalling pathway [1]. Similarly, Neudecker et al. documented that miR-223 overexpression had a protective effect against ALI in a mouse model [23]. Another study indicated that miR-223 contributed to autophagy of mouse pulmonary microvascular endothelial cells and thereby aggravated lung ischaemia-reperfusion injury [24]. Consistently, we demonstrated that miR-223 was downregulated in LPS-treated alveolar epithelial cells in this study. Overexpression of miR-223 reversed LPS-treated oxidative stress and excessive autophagy in A549 cells, indicating that miR-223 has a protective effect against LPS-induced ALI. However, in another study, miR-223 expression was increased in ALI mouse model and ARDS patients [25]. Furthermore, the upregulation of miR-223 inhibited NLRP2 expression and IL-1, which mediated ALI. These contradictory results may be due to individual differences or different experimental models.

Previous studies have mainly focused on the critical role of HMGB1 in the pathogenesis of inflammation [26]; however, a recent study indicated that HMGB2 and HMGB1 may bind to inflachromene, a drug with an anti-inflammatory effect, and suppress microglia-mediated inflammation [27]. Furthermore, HMGB2 was documented to mediate cell autophagy in a previous study. Liu et al. noted that HMGB2 enhanced ROS production in cardiac cells and thereby led to abnormal autophagy [4]. Data from An et al. demonstrated that downregulation of HMGB2 in gastric cancer cells significantly reduced the level of autophagy [28]. Although both miR-223 and HMGB2 are involved in mediating inflammation and autophagy in ALI, the relationship between miR-223 and HMGB2 was not reported. As far as we know, we demonstrated for the first time that miR-223 directly targeted HMGB2 as indicated by the luciferase reporter assay. We further showed that silencing of HMGB2 restored LPS-induced oxidative stress, excessive autophagy and inflammation in A549 cells. In contrast, overexpression of HMGB2 had an opposite effect. These data further confirmed that miR-223 attenuates oxidative stress, autophagy and inflammation in LPS-treated alveolar epithelial cells by targeting HMGB2.

The JNK signalling pathway is a well-accepted cascade involved in numerous cellular functions, including cell proliferation, migration, and apoptosis [29, 30]. Additionally, this signalling pathway was documented to play a critical role in mediating autophagy [31]. The JNK signaling pathway activation during ALI resulted in excessive autophagy in alveolar epithelial cells [7]. Thus, we proceed to investigate whether the JNK signalling pathway is involved in the protective effects of miR-223 against ALI. Knockdown of HMGB2 or miR-223 overexpression inhibited the activation of the JNK signalling pathway in LPS treated alveolar epithelial cells, whereas HMGB2 overexpression had a contrasting effect. Inhibition of the JNK signalling pathway significantly attenuated the protective effects of miR-223 against LPS induced apoptosis, excessive autophagy and inflammation. In summary, we demonstrated that the protective effect of miR-223 on ALI were entirely or at least partially mediated via the HMGB2/JNK axis and that miR-223 may be a novel therapeutic target for ALI treatment. However, in the present study, we focused only on *in vitro* cell-based models to demonstrate the principle purpose. Future studies will focus on demonstrating the correlation among miR-223, HMGB2, oxidative stress and autophagy in response to ALI, especially the effects on autophagy and oxidative stress in ALI mice that knock down miR-233 in previously reported miR-223^−/−^ mice [23, 32].

## Conclusions

In conclusion, this study is the first to report that miR-223 suppresses the autophagy in LPS induced alveolar epithelial cells by inhibiting HMGB2 expression. Based on the importance of the excessive autophagy of alveolar epithelial cells in the pathological process of ALI, miR-223 can potentially be used as a protective factor and offers a new therapeutic strategy for ALI treatment.

## Supplementary Information

**Additional file 1.**

## Data Availability

All data generated or analysed during this study are included in this published article.

## Abbreviations

8-OHdG: 8-hydroxy-2’ -deoxyguanosine
ALI: Acute lung injury
ARDS: Acute respiratory distress syndrome
DCF: Dichlorofluorescein
ELISA: Enzyme-linked immunosorbent assay
HMGB: High-mobility group box
JNK: c-Jun N-terminal kinase
LPS: Lipopolysaccharide
miR223: miRNA-223
MAPK: Mitogen-activated protein kinase
NF-κB: Nuclear factor-κB
PBS: Phosphate-buffered saline
qPCR: Quantitative polymerase chain reaction
ROS: Reactive oxygen species
