# Correction to: Downregulation of miR-223 promotes HMGB2 expression and induces oxidative stress to activate JNK and promote autophagy in an in vitro model of acute lung injury

**DOI:** 10.1186/s12950-021-00297-1

**Published:** 2021-12-07

**Authors:** Hao-Yu Tan, Bei Qing, Xian-Mei Luo, Heng-Xing Liang

**Affiliations:** 1 Department of Cardio-vascular Surgery, the Second Xiangya Hospital of Central South University, No.139 Middle Renmin Road, Hunan Province 410011 Changsha, People’s Republic of China; 2 Department of Thoracic Surgery, the Second Xiangya Hospital of Central South University, No.139 Middle Renmin Road, Hunan Province 410011 Changsha, People’s Republic of China

**Correction to:** ***J Inflamm*** **18, 29 (2021).**

**https://doi.org/10.1186/s12950-021-00295-3**

Following publication of the original article [1], it was reported that there was an error in the affiliations. Affiliation 1 was erroneously assigned to all authors instead of only to Hao-Yu Tan.

The correct affiliation information is given in this Correction and the original article [1] has been updated.

## References

[1] Tan HY, Qing B, Luo XM, Liang HX (2021). Downregulation of miR-223 promotes HMGB2 expression and induces oxidative stress to activate JNK and promote autophagy in an in vitro model of acute lung injury. J Inflamm.

