# Correction: Downregulation of miR-223 promotes HMGB2 expression and induces oxidative stress to activate JNK and promote autophagy in an in vitro model of acute lung injury

**DOI:** 10.1186/s12950-024-00424-8

**Published:** 2025-01-06

**Authors:** Hao-Yu Tan, Bei Qing, Xian-Mei Luo, Heng-Xing Liang

**Affiliations:** 1 Department of Cardio-Vascular Surgery, the Second Xiangya Hospital of Central South University, No.139 Middle Renmin Road, Hunan Province, 410011 Changsha, People’s Republic of China; 2 Department of Thoracic Surgery, the Second Xiangya Hospital of Central South University, No.139 Middle Renmin Road, Hunan Province, 410011 Changsha, People’s Republic of China

**Correction** **:** **J Inflamm 18, 29 (2021)**

**https://doi.org/10.1186/s12950-021-00295-3**

Following publication of the original article [1], the authors proposed to clarify some figures in the article.

To describe MTT results more accurately, the authors revised the ordinate name of MTT result from Cell viability (OD 490nm) to “**OD value (490nm)**” in the y-axis of **Figure 1A**. Meanwhile, to describe the flow cytometry results more accurately, the authors revised the ordinate name of the statistical results from Apoptosis rate (%) to “**Annexin V+/PI- and Annexin V+/PI+ cells**” in the y-axis of **Figure 1D, 2C, 4D, 4K and 5D**.

The correct figures are as follows:

Fig 1


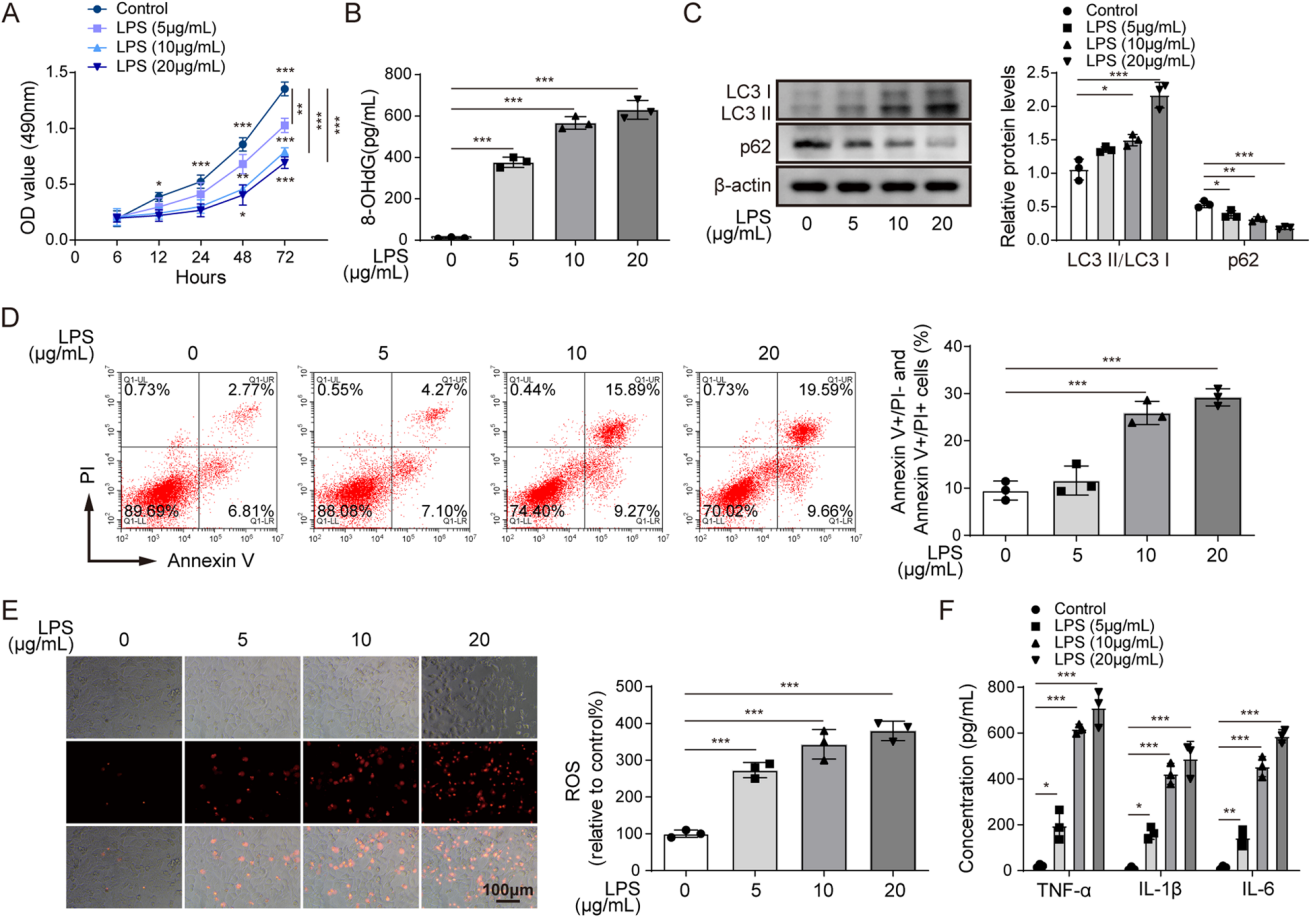


Fig 2


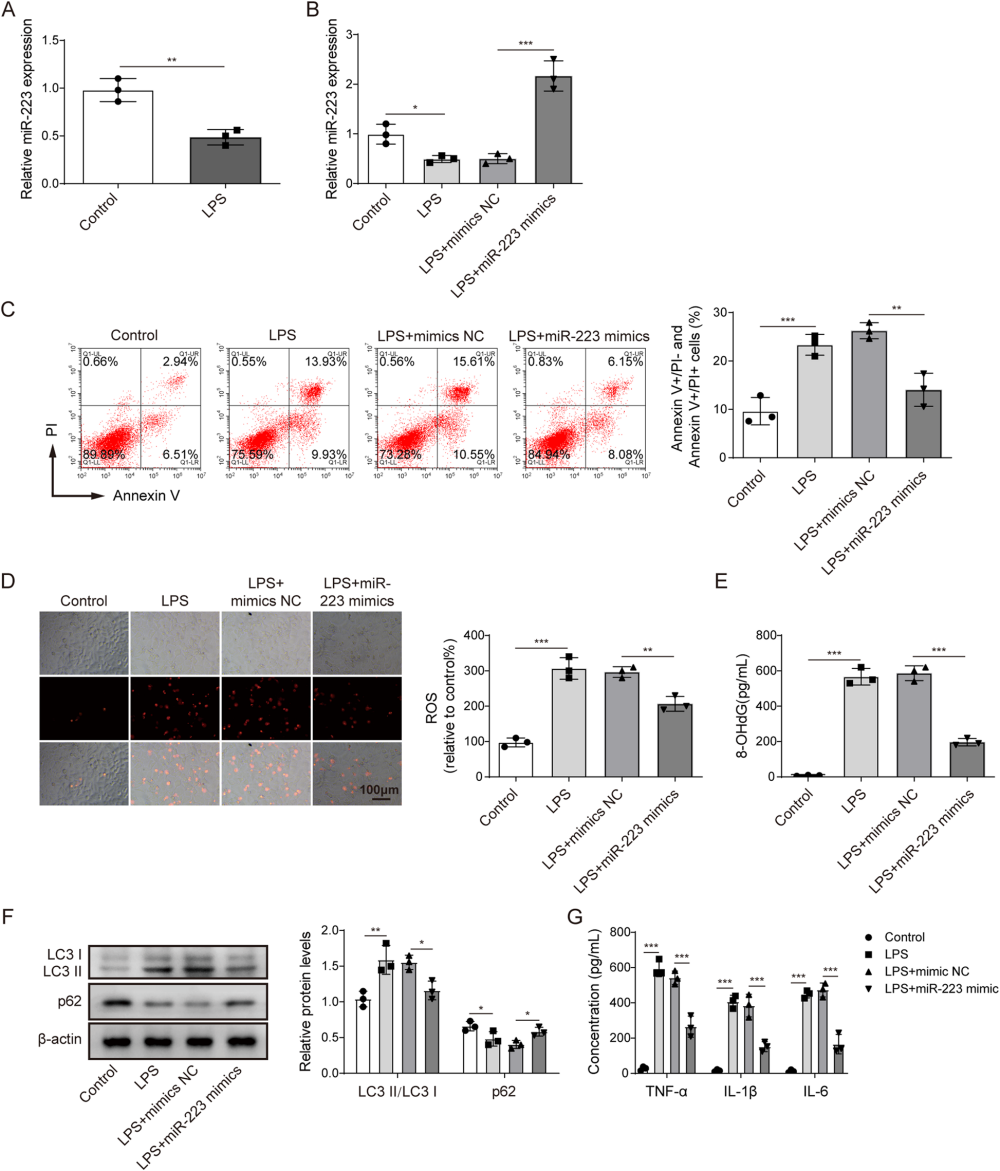


Fig 3


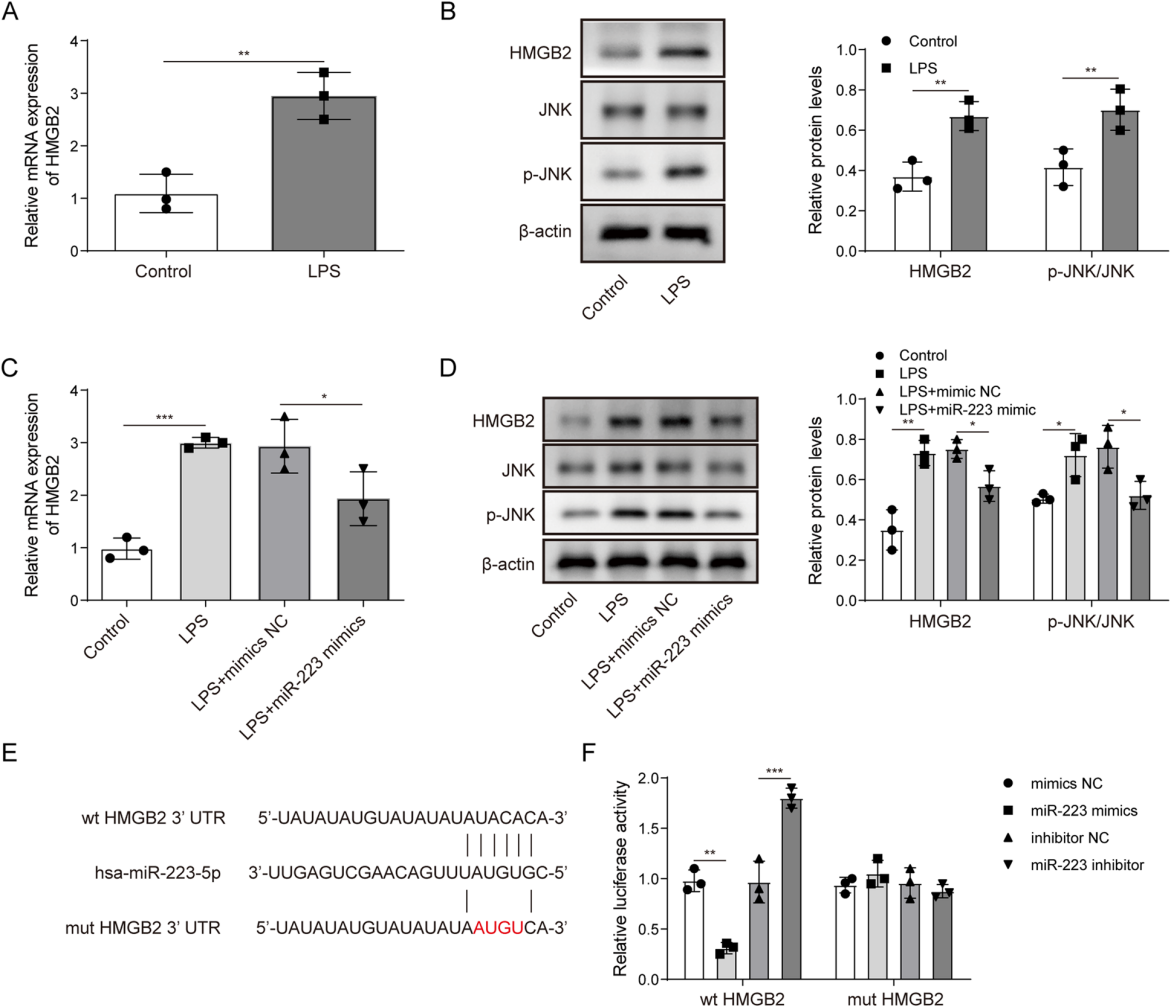


Fig 4


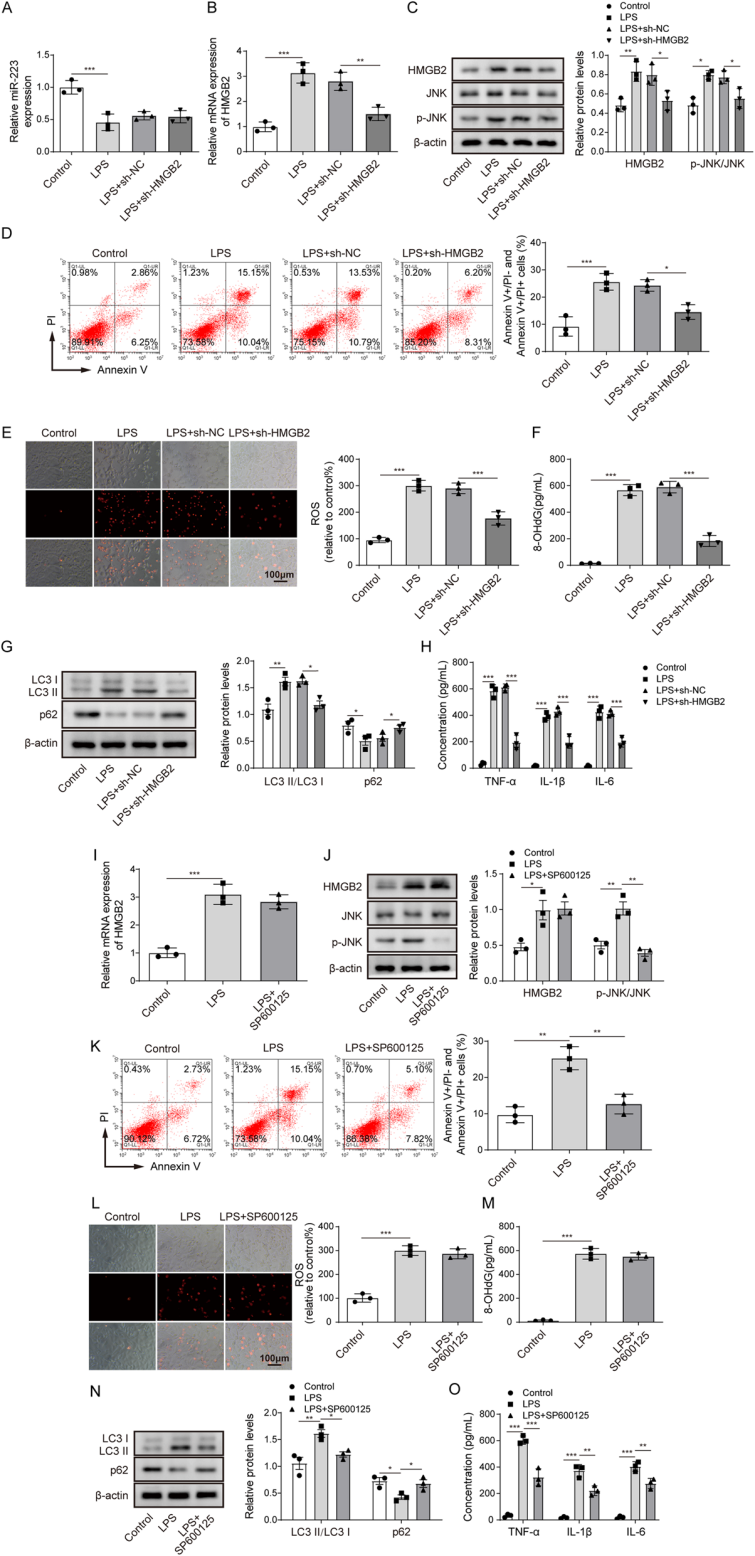


Fig 5


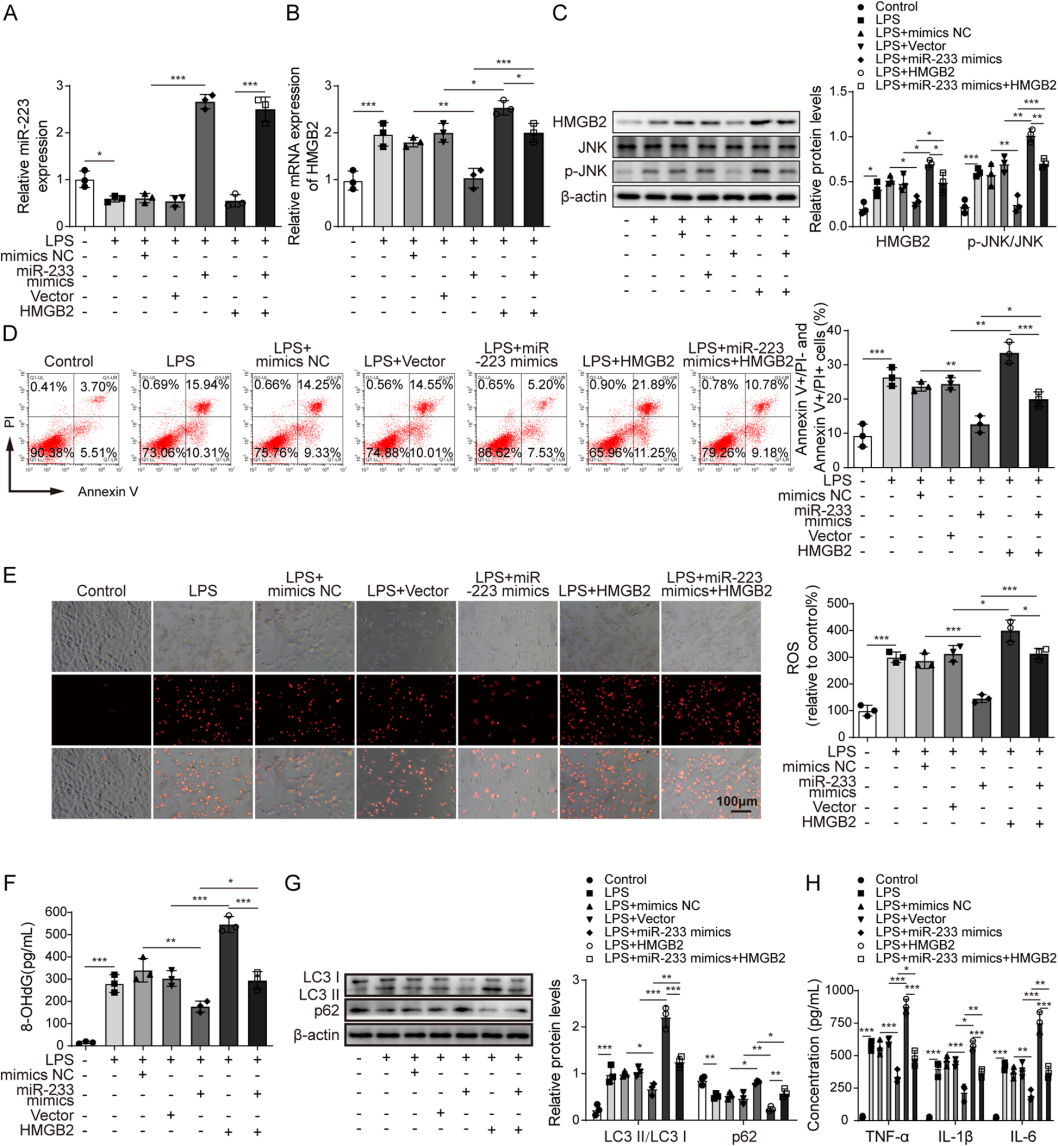


The original article [1] has been updated.
