# Supplementary figures and images for: Downregulation of miR-223 promotes HMGB2 expression and induces oxidative stress to activate JNK and promote autophagy in an in vitro model of acute lung injury
Source: J Inflamm (Lond). 2021 Nov 3;18:29. doi: 10.1186/s12950-021-00295-3 (PMC8565047; doi:10.1186/s12950-021-00295-3)

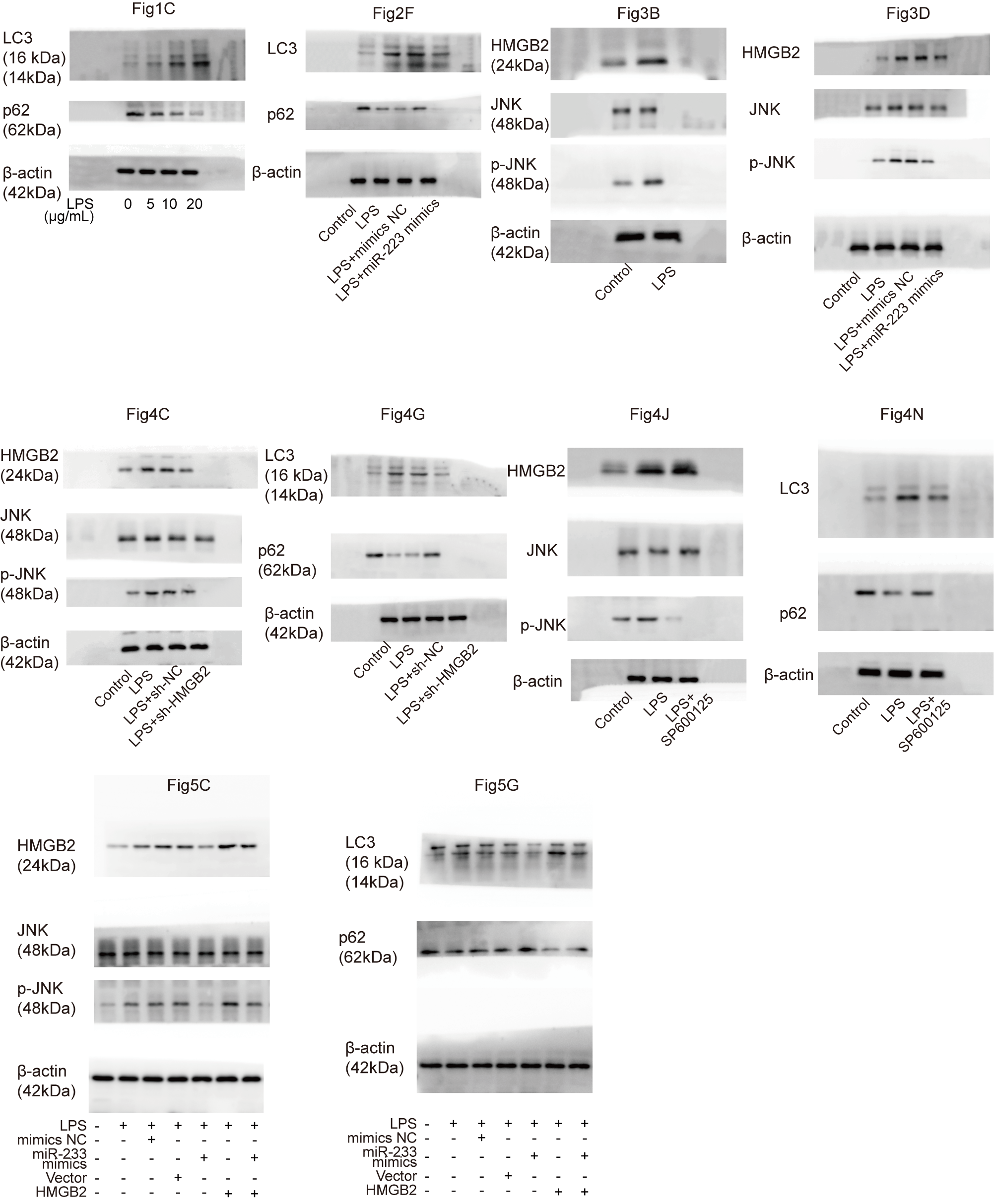

Supplement: Supplementary file 1 — Additional file 1. [file 12950_2021_295_MOESM1_ESM.tif]
